# X-ray photon attenuation measurement as a technique for monitoring liquid composition

**DOI:** 10.1155/S1463924685000190

**Published:** 1985

**Authors:** Sandra Everett, David J. Malcolme-Lawes

**Affiliations:** Chemistry Department King's College London Strand London WC2R 2LS UK


Journal of Automatic Chemistry/*Journal of Clinical Laboratory Automation*, Vol. 7, No. 2 (Apr–Jun 1985), pp. 85–89

### X-ray photon attenuation measurement as a technique for monitoring liquid composition

**Sandra Everett and David J. Malcolme-Lawes**
*Chemistry Department, King's College London, Strand, London WC2R 2LS*

### Introduction

In a recent paper [1] the authors reported some results of experiments on monitoring the attenuation of a collimated beam of 60 keV gamma photons by a liquid mixture flowing through a large diameter (10 cm) pipe. It was found that the measurement of the attenuation offered a technique for composition monitoring of substantial scale liquid-mixture flows. However, the energy of the radiation was too large to be useful for monitoring attenuation in smaller-scale systems found in many industrial environments. In this paper an extension of the previous work is reported in which lower energy X-ray photons have been used. Because these photons are less energetic, they are less penetrating; smaller path lengths can be used and the system is more appropriate for monitoring liquid flows in smaller-scale industrial systems.

When X-rays pass through matter they become scattered and absorbed. At the energies of interest here (<100 keV), the most important effects are those produced by the photoelectric and Compton processes. If a collimated beam of mono-energetic photons is attenuated to an intensity $I$ from an initial intensity $I_0$ in passing through a layer of material of path length $x$, then according to the Bouger-Beer-Lambert law:

$$I_x = I_0 \exp(-ux) \qquad (1)$$

where $u$ is the linear attenuation coefficient of the material concerned.

The linear attenuation coefficient of a mixture of materials [2] is given by:

$$u = \mathrm{p}\sum_i g_i\,(u/p)_i \qquad (2)$$

where $g_i$ is the mass fraction of component $i$, $(u/p)_i$ is the mass absorption coefficient of component $i$, and $p$ is the density of the mixture.

The mass absorption coefficient $(u/p)$ [3] is given by:

$$(u/p) = \sum_j g_j\,(u/p)_j \qquad (3)$$

where $(u/p)_j$ is the mass absorption coefficient of element $j$, and $g_j$ is the mass fraction of element $j$.

The mass absorption coefficient of an element $(u/p)$ [3] is given by:

$$(u/p) = o(\mathrm{total})*(\mathrm{N}/M) \qquad (4)$$

where N is the Avogadro constant, $M$ is the atomic weight and $o$(total) is the total cross-section for the removal of photons from a collimated, mono-energetic beam.

Using equations (1)–(4) it is possible to calculate the expected transmission through a mixture of materials using tabulated or theoretical values for the atomic scattering cross-sections, providing that the density is known [4]. These calculations are normally performed for systems under experimental investigation, and the theoretical values are plotted along with experimental results in the graphs presented below. It is important to note that some theoretical values are expected to contain substantial absolute errors arising from the use of tabulated data for hydrogen – which appears to be unsuitable for the present purpose. Nevertheless, theoretical values are useful because they allow the change in attenuation with sample composition to be estimated with reasonable confidence, and thus provide a means of predicting the precision with which composition measurements may be made. For this reason, the theoretical results are included on the graphs and no attempt has been made to adjust published atomic scattering cross-sections to force better absolute agreement.

### Experimental

The apparatus used for monitoring the absorption of X-rays by flowing mixtures is shown schematically in figure 1. The photon source was a variable energy X-ray source (Amersham International PLC, code number AMC.2084). The primary source consisted of a sealed, ceramic source of 10 mCi Americium-241. This annular source irradiates a selected target disc with 60 keV gamma photons, exciting characteristic X-ray emission from the target material. The primary source surrounds a 4 mm diameter X-ray emission aperture in the stainless-steel assembly while the targets are mounted on a rotary holder, which may be moved to select an X-ray energy between 8 and 44 keV. For the experiments reported below two targets were used: (1) the silver target which emits characteristic Ka radiation at 22·10 keV; and (2) the barium target which emits characteristic Ka radiation at 32·06 keV.

The semi-collimated beam of X-rays from the variable energy source produced a count rate of approximately $3*10^3\,s^{-1}$ in the absence of a sample. (More intense single energy sources are available.) The beam passed normally through a glass flow-cell of path length 2 cm. The liquid was pumped through the flow cell using a peristaltic pump (Watson-Marlow 501-U model). The attenuated beam was monitored by a microcomputer (CBM 8032) using a NaI(Tl) scintillation detector probe (type 5·42 Mini Instruments Ltd) via a 24-bit pulse counter interface unit. A second 24-bit pulse counter was used to

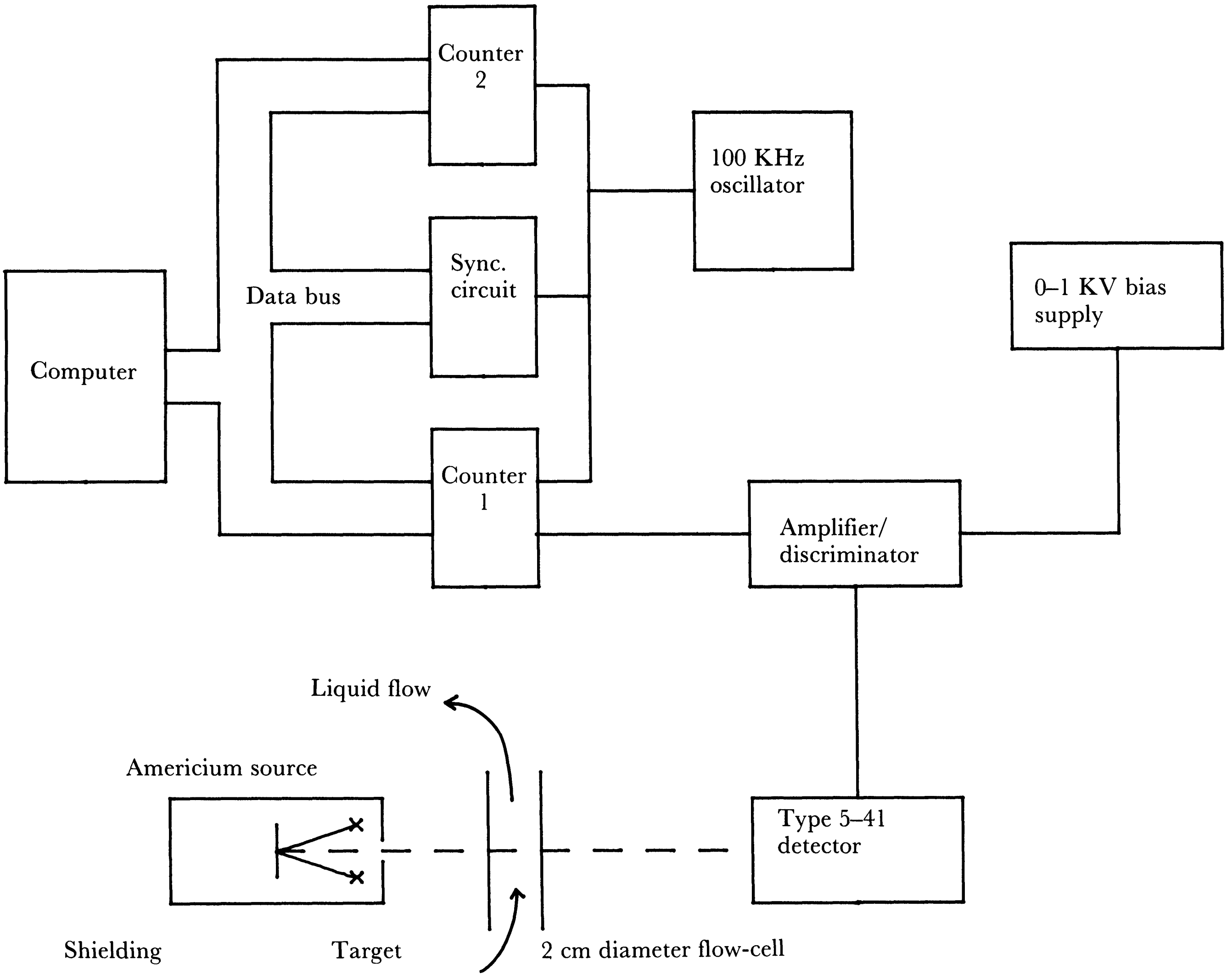

*Figure 1. Representation of the experimental system used for monitoring the intensity the X-ray photon beam after passing through a liquid mixture.*

count pulses from a 100 kHz clock oscillator. The microcomputer evaluated the count rate and percentage transmission of the incident X-ray beam, either for a predetermined statistical error limit, or for a preset counting period in seconds. A detailed description of the interface used is given in a 1984 issue of *Laboratory Microcomputer* [5].

The samples used in these experiments consisted of total volumes of 0·5–1 litres and were made up to an estimated accuracy of 1%. Results were obtained for several binary mixtures including organic liquids (hexane/decane) and ionic solutions (aqueous ferrous sulphate). Results were also obtained for some mixtures which were of more obvious commercial significance, such as ethanol/water mixtures, and some for which the elemental composition of the components was not known and theoretical comparisons could not be made. These samples were a fat in water emulsion (cream and water mixtures), and a silicone resin (a commercial water-proofing compound) in white spirit.

### Results and discussion

The results are shown in figures 2–6, with percentage photon transmission plotted as a function of sample composition in each case. All the results have a quadratic curve drawn through them, the best-fit equation for which was calculated from the experimental data using the method of polynomial regression. In all cases, the error bars shown represent the standard error in the mean for 10 readings, each reading being accurate to a statistical error limit of less than 0·5%. This represents a total counting time of between 100 and 500 s (depending on the composition of the mixture). Of course, the same error limits could have been achieved with much shorter counting times by using a more intense and better collimated source. In figures 2–4 the theoretical values of the transmission through a 2 cm path-length of the sample are also shown (the crossed points which form the lower line in each case). The atomic attenuation coefficients used to calculate the theoretical linear attenuation coefficients are shown in table 1. As the absolute atomic

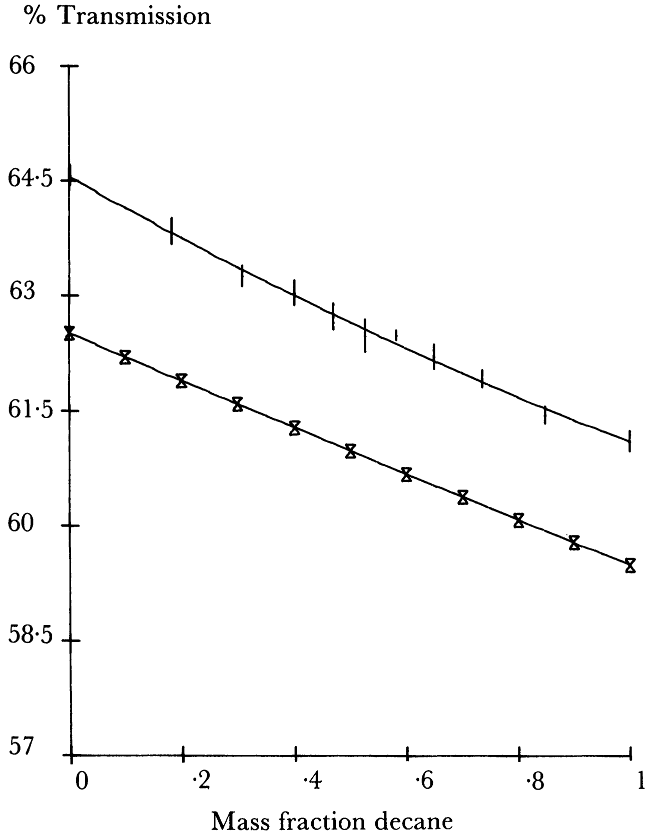

*Figure 2. Silver (22·10 keV) X-ray transmission as a function of mixture composition for decane/hexane mixtures.*

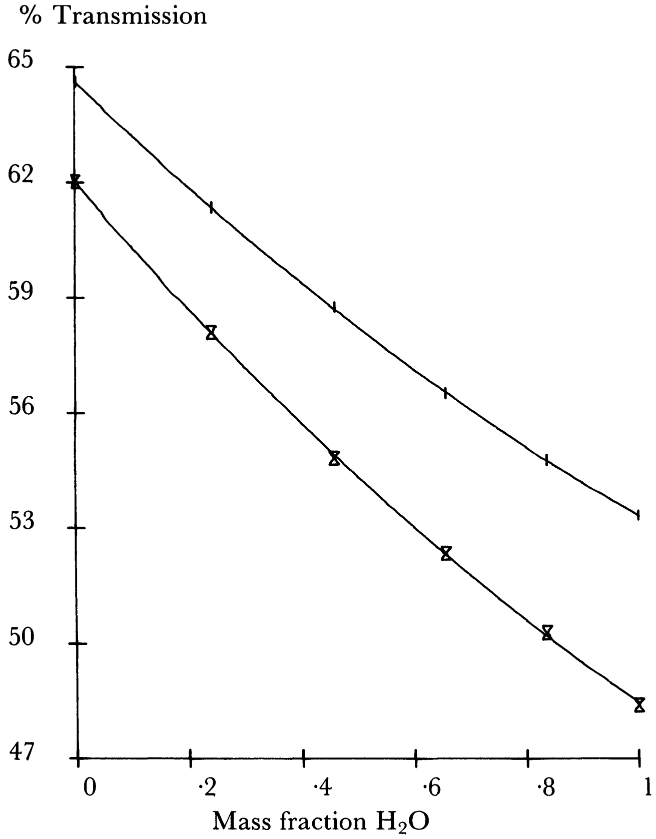

*Figure 3. Barium (32·06 keV) X-ray transmission as a function of mixture composition for ethanol/water mixtures. Note that the theoretical curve is based on attenuation coefficients at 30 keV.*

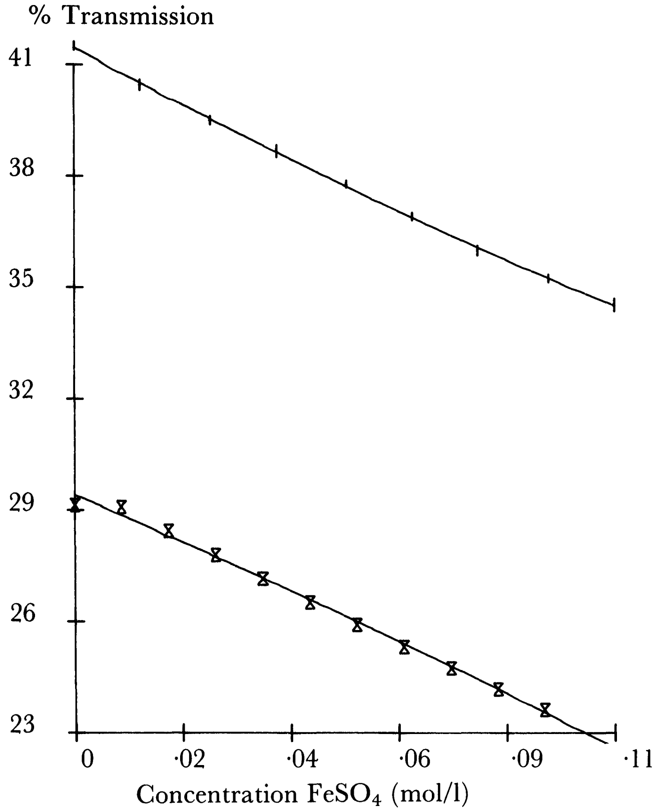

*Figure 4. Silver (22·10 keV) X-ray transmission as a function of mixture composition for $FeSO_4$ solutions.*

composition of the white spirit, silicone resin and cream was not known, the theoretical linear attenuation coefficients could not be calculated for the systems shown in figures 5 and 6.

Figure 2 shows the results for two totally miscible organic liquids (hexane and decane) using the silver target. Because these two liquids are similar in composition and density, their linear attenuation coefficients are of comparable magnitude: consequently there is only a small change in transmission over the whole range of compositions. In this case the estimation of the composition of an hexane/decane mixture under the conditions of these experiments would result in an uncertainty of the order of ± 0·05 in the decane mass fraction. Of course this figure could be improved by the use of a higher intensity source or by measuring for a longer time period. The present result indicates the limitation of the technique for monitoring two materials which are very similar in their atomic constitution. It is clear that the theoretical transmissions are consistently lower than the experimentally measured values by about 2%, although the predicted change in transmission with composition (i.e. the slope of the graph) is in much closer agreement with the experimental finding.

A somewhat more sensitive variation in transmission is found for barium X-rays passing through ethanol/water mixtures as shown in figure 3. Although the energies are different, the transmission of ethanol is very similar to that of the hexane in figure 2 (approximately 64·5%), while the transmissions of water (53·4%) and decane (61·5%) are significantly different. Under the conditions of these experiments the composition of an ethanol/water

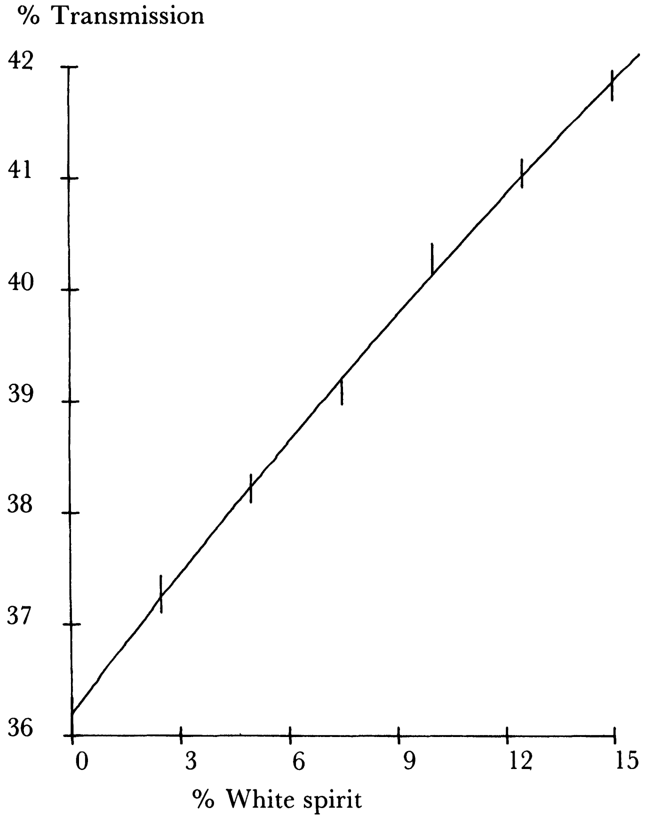

*Figure 5. Barium (32·06 keV) X-ray transmission as a function of mixture composition for white spirit/silicone resin mixtures.*

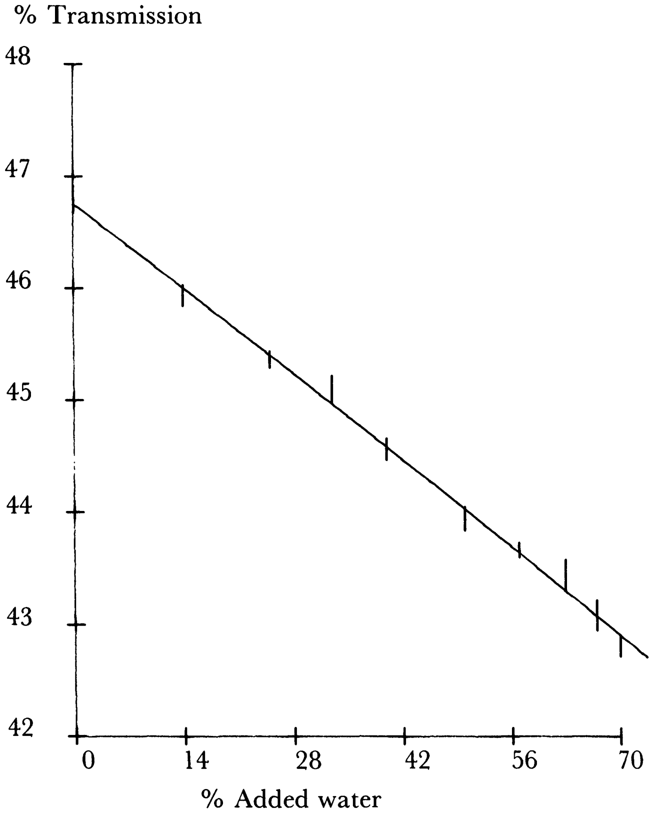

*Figure 6. Silver (22·10 keV) X-ray transmission as a function of mixture composition for cream/water mixtures.*

*Table 1. Attenuation coefficients.*

| (i) Atomic attenuation coefficients[4] | | |
|---|---|---|
| | $u/p/cm^2 g^{-1}$ | |
| Element | 22·1 keV | 30·0 keV |
| H | 0·3363 | 0·3570 |
| C | 0·3537 | 0·2482 |
| O | 0·6479 | 0·3634 |
| S | 4·816 | |
| Fe | 19·31 | |
| **(ii) Linear attenuation coefficients used for compounds** | | |
| | $u/cm^{-1}$ | |
| Sample | 22·1 keV | 30·0 keV |
| Water | 0·6164 | 0·3626 |
| Ethanol | | 0·2387 |
| Hexane | 0·2349 | |
| Decane | 0·2596 | |
| 0·05M $FeSO_4$ | 0·8005 | |

mixture may be estimated with an uncertainty of better than ±0·02 in the water mass fraction. Again, however, the theoretical transmissions (calculated using attenuation coefficients of 30 keV photons) are consistently lower than the experimentally measured values. (The slight difference in slope between the experimental and theoretical lines probably arises from the slightly different X-ray energies used. Theoretical values for 32·06 keV were not available.)

Figure 4 shows the results for aqueous solutions of ferrous sulphate over the concentration range up to about 0·1 M, using the silver X-rays. Even for these fairly dilute solutions there is a substantial change in the transmission of the X-ray beam with concentration. This is due to the fact that iron has a much larger atomic attenuation coefficient than any of the other elements involved at this energy, so that a small change in the iron content of the mixture produces a large change in the attenuation of the beam. In this case precisions of the order of ±0·001 M in estimated iron concentration are obtained under the conditions of these experiments. The theoretical transmission in this case is substantially lower than the experimental value at all compositions, although, again, the slope of the experimental and theoretical curves agree quite well. The theoretical curves shown in figures 2–4 are based on the atomic attenuation coefficients given by McMaster *et al.* [4] The differences between the experimental and theoretical curves are probably due in large part to the inaccuracy of the atomic attenuation coefficient of hydrogen; empirical adjustment of this quantity gives a much better agreement between the theoretical and experimentl curves.

Figures 5 and 6 show the results for mixtures whose absolute atomic composition is not known precisely, but are the type of mixtures which are of considerable industrial importance and for which alternative composition monitoring techniques are less readily available. The barium target was used for the measurements on the silicone resin in white spirit, and figure 5 shows the results for white spirit addition up to 15% v/v (the range which may be encountered in commercial water-proofing 'concentrate'). In this case the white spirit content may be estimated with a precision of ±1% under the conditions of these experiments. The silver X-rays were used for measurements on the cream/water mixtures (figure 6), and the cream in this case was double cream. As the 'pure' cream contained water, the measurements were made as a function of added water, and the precision with which the amount of added water could be estimated was approximately ±2%.

These results demonstrate that the measurement of X-ray attenuation offers a useful technique for monitoring the variation in composition of a binary liquid mixture flow, and for determining the composition of an unknown mixture given a calibration graph. While absolute values of the attenuation of liquid mixtures calculated from tabulations of atomic scattering cross section may contain substantial errors, it would appear that changes in attenuation with composition are adequately predictable. While the measurement time needed to obtain the precision reported may appear rather long, it is important to remember that these experiments have been performed with a relatively low intensity X-ray source, as is prudent for an experimental apparatus in which the source is manipulated frequently. There is no reason why sources with intensities at least two orders of magnitude larger than that used in this work should not be used with a similar detector system for monitoring liquid flow in a more dedicated system. This, in turn, would result in reductions of the measurement time to levels more acceptable for industrial plant.

### Acknowledgements

This work was supported by British Petroleum. The authors wish to thank the Agrement Board for the provision of silicone fluid samples. David Malcolme-Lawes is a Royal Society Research Fellow in the Physical Sciences.

### References

1. Everett, S. and Malcolme-Lawes D. J., *Journal of Automatic Chemistry*, **6**, 2 (1984).
2. *International Tables for X-ray Crystallography.* Volume IV (The Kynoch Press, 1968).
3. Hubbell, J. H., *Radiation Research*, **70** (1977), p. 58.
4. McMaster, W. H., *et al., US Atomic Energy Commission Report UCRL-50174* (1969).
5. Everett, S. and Malcolme-Lawes, D. J., *Laboratory Microcomputer* (1984).

### SCIENCE AND MANAGEMENT FOR THE FUTURE

*American Association for Clinical Chemistry: Atlanta, Georgia (21 to 26 July 1985)*

The AACC's 37th national meeting will consist of symposia, scientific workshops, roundtables, poster sessions and an exhibition. Symposia include:

**Use of laboratory computers as management tools**
(Developed and organized by the AACC Laboratory Information Systems Division)
Chairman: Kenneth L. Blick
The integration of the laboratory computer into the testing process
Computerization of the laboratory office
The use of the hospital information system in the integration and management of health-care delivery
The use of computer-generated work-loads as a tool for laboratory staffing

**Optimal utilization of the clinical laboratory for clinical decision making**
Chairman: Alex A. Pappas
How and why do physicians order laboratory tests?
Algorithms in clinical laboratory diagnosis
Artificial intelligence in clinical laboratory decision making
Implementation via information transfer systems

Proposed workshop topics are:
Clinical and laboratory assessment of nutritional status
Clinical laboratory management
Methodology and interpretation in toxicology
Physician office-based laboratory testing
Clinical use of ion selective electrodes
Computer aided interpretation of laboratory data
Topics in animal clinical chemistry
Prospective payment systems, DRGs and the clinical laboratory
Environmental toxicology
Pediatric adolescent clinical chemistry
Laboratory data and information systems
Diagnostic virology and infectious diseases testing using immunoassays
Legal aspects of clinical laboratory practice
Recent advances in the application of chromatographic techniques to the clinical laboratory
New approaches to immunofixation and immunoelectrophoresis.

*More information from Deborah A. Woodcock, Director of Meetings, American Association for Clinical Chemistry, 1725 K Street, N.W., Suite 1010, Washington, D.C. 20006. Tel.: 202 857 0717.*

